# Integrated Gene Expression Profiling Analysis Reveals Potential Molecular Mechanisms and Candidate Biomarkers for Early Risk Stratification and Prediction of STEMI and Post-STEMI Heart Failure Patients

**DOI:** 10.3389/fcvm.2021.736497

**Published:** 2021-12-10

**Authors:** Jing Xu, Yuejin Yang

**Affiliations:** ^1^State Key Laboratory of Cardiovascular Diseases, Fuwai Hospital and National Center for Cardiovascular Diseases, Beijing, China; ^2^Peking Union Medical College, Chinese Academy of Medical Sciences, Beijing, China

**Keywords:** acute myocardial infarction, heart failure, microarray, machine learning, biomarker

## Abstract

**Objective:** To explore the molecular mechanism and search for the candidate differentially expressed genes (DEGs) with the predictive and prognostic potentiality that is detectable in the whole blood of patients with ST-segment elevation (STEMI) and those with post-STEMI HF.

**Methods:** In this study, we downloaded GSE60993, GSE61144, GSE66360, and GSE59867 datasets from the NCBI-GEO database. DEGs of the datasets were investigated using R. Gene ontology (GO) and pathway enrichment were performed *via* ClueGO, CluePedia, and DAVID database. A protein interaction network was constructed *via* STRING. Enriched hub genes were analyzed by Cytoscape software. The least absolute shrinkage and selection operator (LASSO) logistic regression algorithm and receiver operating characteristics analyses were performed to build machine learning models for predicting STEMI. Hub genes for further validated in patients with post-STEMI HF from GSE59867.

**Results:** We identified 90 upregulated DEGs and nine downregulated DEGs convergence in the three datasets (|log_2_FC| ≥ 0.8 and adjusted *p* < 0.05). They were mainly enriched in GO terms relating to cytokine secretion, pattern recognition receptors signaling pathway, and immune cells activation. A cluster of eight genes including *ITGAM, CLEC4D, SLC2A3, BST1, MCEMP1, PLAUR, GPR97*, and *MMP25* was found to be significant. A machine learning model built by *SLC2A3, CLEC4D, GPR97, PLAUR*, and *BST1* exerted great value for STEMI prediction. Besides, *ITGAM* and *BST1* might be candidate prognostic DEGs for post-STEMI HF.

**Conclusions:** We reanalyzed the integrated transcriptomic signature of patients with STEMI showing predictive potentiality and revealed new insights and specific prospective DEGs for STEMI risk stratification and HF development.

## Introduction

Acute myocardial infarction (AMI) is a consequence of rupture or erosion of a vulnerable, lipid-laden, chronic atherosclerotic coronary plaque, resulting in acute interruption of myocardial blood flow and ischemic myocardial necrosis, which remains a common cardiac emergency incidence with substantial morbidity and mortality worldwide (1, 2). Concurrently, low- and middle-income countries now cover more than 80% of deaths from cardiovascular disease worldwide, which contributes to the societal burden, as assessed by impaired disability-adjusted life-years (3, 4). The current diagnostic evaluation for the presence of AMI relies on troponin or creatine kinase MB-fraction assays in addition to an electrocardiogram (ECG), which detects necrotic cardiomyocytes (5). However, it has been recognized for decades that most atherosclerotic lesions underlying AMI are only partial luminal narrowing prior to acute plaque rupture and not obstructing the coronary blood flow (6–8). Consequently, the inability to accurately and temporally predict the occurrence of AMI impairs our capability to further improve patient outcomes.

Acute myocardial infarction has traditionally been classified on the basis of the presence or absence of ST-segment elevation (STEMI or non-STEMI) on the ECG. It is pertinent to note that a totally occlusive thrombus typically leads to STEMI and develops transmural or Q-wave MI, whereas most patients with non-STEMI have a partial occlusion or occlusion in the presence of collateral circulation, develop subendocardial, non-transmural, or non-Q-wave MI (2). However, STEMI is not only a major killer in both elderly and non-elderly (age <65 years) patients (9), but survivors of acute STEMI are prone to develop progressive ventricular remodeling and dysfunction that leads to heart failure (HF) (10–12).

While advances in the contemporary management of STEMI have improved rates of short-term survival, the subsequent progression of HF is emerging as a prominent cause of long-term outcomes, despite sustained potency of the infarct-related artery, by the successful percutaneous coronary intervention (PCI) (13). Moreover, recommended HF-associated biomarkers, including B-type natriuretic peptide (BNP) and N-terminal probrain natriuretic peptide (NT-proBNP), lack specificity that they can also exhibit elevated levels in patients with congestive HF, renal failure, primary aldosteronism, and thyroid disease (14–16). There are, therefore, novel robust biomarkers with predictive potentiality for screening the chronic ischemic preconditioning and the occurrence of STEMI, and also the development of post-STEMI HF remains a crucial target for scientific advancement in cardiovascular diseases.

Next generation sequencing (NGS) technology is the driving force for genome-wide gene expression profiling, and transcriptome analysis *via* indispensable bioinformatics approaches has been extensively used for obtaining novel insights into mechanisms underlying the development of diseases and identifying the potential biomarkers (17, 18). In the present study, we performed an integrated gene expression profiling analysis and applied a machine-learning algorithm to investigate the shared molecular patterns and identify prognostic/diagnostic differentially expressed genes (DEGs) associated with STEMI and post-STEMI HF, and detectable in the peripheral blood of patients, which may contribute to the early warning and optimized risk stratification of AMI.

## Materials and Methods

### Data Resources

Microarray data profiles of GSE60993, GSE61144, GSE66360, and GSE59867 were downloaded from the Gene Expression Omnibus database (https://www.ncbi.nlm.nih.gov/geo/), an international public repository from the National Center for Biotechnology Information (NCBI) that provides free access to full sets of genome data submitted by the research community (19). The GSE60993 dataset, tested on GPL6884 based on Illumina HumanWG-6 v3.0 Expression BeadChip and the GSE61144 dataset, tested on GPL6106 based on Sentrix Human-6 v2 Expression BeadChip, were the blood transcriptome-based signatures of patients with the acute coronary syndrome (ACS) and control participants (20). Patients diagnosed with other subtypes of ACS, including NSTEMI and UAP, were eliminated from the subsequent analysis. The GSE66360 dataset comprised the microarray profiles of the circulating endothelial cells (CEC) from patients experiencing STEMI and healthy cohorts, tested on GPL570 *via* Affymetrix Human Genome U133 Plus 2.0 Array (21). Additionally, the GSE59867 dataset contains the gene expression profiles of peripheral blood mononuclear cells from nine patients with post-STEMI HF and eight non-HF controls divided on the basis of plasma NT-proBNP level and left ventricular ejection fractions (LVEF) (22). After the platform descriptions matrix files were downloaded, the gene probe was matched to the corresponding official gene symbol. For the situation that multiprobes to one gene, we retained the probe which shows the most significant gene expression value (adjusted *p*-value) after deleting the non-mRNA probes. The following procedures were processed based on the matched matrix files.

### Screening and Identification of Significant DEGs

We used the *limma* package to screen DEGs in selected samples from GSE60993, GSE61144, and GSE66360, respectively based on the R platform (R-project.org). The data of the three datasets were all normalized using quantile normalization. The value was log2 transformed and the genes with detection *p* > 0.1 were removed at all arrays after quantile normalization. Fold change (FC) was obtained by calculating the ratio of the expression of each gene between STEMI and control samples in every dataset. Logarithmic operations with 2 as the base number were utilized to make easier calculations and more scientific comparisons. For a more comprehensive identification, genes with |log_2_FC| ≥ 0.8 were considered as DEGs, and statistical differences were defined as threshold values by adjusted *p* < 0.05, corrected by the *Benjamini-Hochberg* method. DEGs with log_2_FC <0 were considered downregulated, whereas those with log_2_FC > 0 were considered upregulated. The DEG results were further validated *via* GEO2R, the online R-based web application supported by the GEO database (19). Considering the microarray profiles from different datasets were all collected from peripheral blood samples, including total blood RNA and CEC-derived RNA, we selected the DEG signature convergence in the three datasets for further analysis, which also reflected the tissue–cell relationship.

### Functional Annotation of DEGs

Gene ontology (GO) analysis refers to determining and describing the biological characteristics of genome or transcriptome data in different databases by standard expression terms (23). The cellular component (CC), molecular function (MF), and Kyoto Encyclopedia of Genes and Genomes (KEGG) pathway annotations of the 99 common DEGs were conducted *via* Database for Annotation, Visualization and Integrated Discovery (DAVID, http://david.ncifcrf.gov/, version 6.8), a web-based bioinformatics resources with tools for the functional interpretation of large-scale gene datasets (24). *Homo sapiens* were selected to limit the annotation of the species. The biological process (BP) annotation was conducted and visualized by ClueGO (version 2.5.7) and CluePedia (version 1.5.7) tool kits, which can decipher functionally grouped GO and pathway annotation networks with a hypergeometric test and extract representative functional correlations among pathways *via* Cytoscape software (version 3.8.2) (25–27). A *p* < 0.05 was considered statistically significant.

### Protein Interaction and Module Analysis

To analyze the internal connection between all the selected DEGs, a Search Tool for the Retrieval of Interacting Genes (STRING, http://string-db.org/, version 11.0) was performed to predict and construct the protein-protein interaction (PPI) network (28). STRING is a biological database that contains information from multisources, including text mining in PubMed, experimental/biochemical evidence, coexpression, and database association to provide integrated functional interactions between proteins, which may provide novel insights into the mechanisms of diseases (29, 30). The DEGs list was uploaded, and *Homo sapiens* was selected as the organism. To further narrow the candidate gene field, a confidence level of high confidence (0.70) was assessed. Then, PPI networks were visualized using the Cytoscape software. The plug-in Molecular Complex Detection (MCODE, version 1.6.1) algorithm, an automated kit based on the topology to find densely connected regions as molecular complexes or clusters in large PPI networks, was used to screen the hub genes (31). The MCODE parameters criteria were set by default as follows: degree cut-off = 2, node score cut-off = 0.2, Max depth = 100, and *k*-score = 2.

### Prediction Model Analysis by Logistic LASSO Regression

The *glmnet* package in R software was conducted to calculate and select the linear models and preserve valuable variables by the LASSO logistic regression algorithm (32). With the LASSO method, coefficients of unimportant variables are dropped exactly to zero, while important variables are retained to reduce the overfitting (33). The expression levels of hub genes and the diagnosis of 99 samples were obtained from the probe-matched matrix file of GSE66360, according to its largest number of samples among the selected datasets, and the samples were randomly assigned to a training or testing set in approximately a 2:1 ratio. We used a binomial distribution variable in the LASSO classification because of the binary output variable in the processed data, as well as used the 1 standard error of the minimum criteria (the 1-SE criteria) lambda value to build the model with good performance but the least number of variables for 5-fold cross-validation. The displaying of receiver operating characteristics (ROC) analysis and the calculation of the area under the curve (AUC) were conducted by the *pROC* package in R (34). Thus, we investigated the feasibility of the cluster genes in prediction *via* the AUC value.

### Hub Genes Validation in a Post-STEMI HF Cohort

Microarray profiles of patients with post-STEMI HF (*n* = 9) and patients with non-HF (*n* = 8) at four time points, admission, discharge, after 1 month, and after 6 months, were collected from the GSE59867 dataset (22). The expression values of hub genes were screened in these samples and compared between patients with post-STEMI HF and patients with non-HF (*Wilcoxon rank-sum test*). To identify the discriminatory power of each selected biomarker correlated to HF progression which may exert clinical prognostic feasibility, a ROC curve was constructed and the AUC with 95% confidence interval was calculated. The cutoff value for each marker was defined as the marker fold change that corresponds to the point on the ROC curve closest to the point (0, 1).

## Results

### Screening and Identification of DEGs

**Figure 1:**
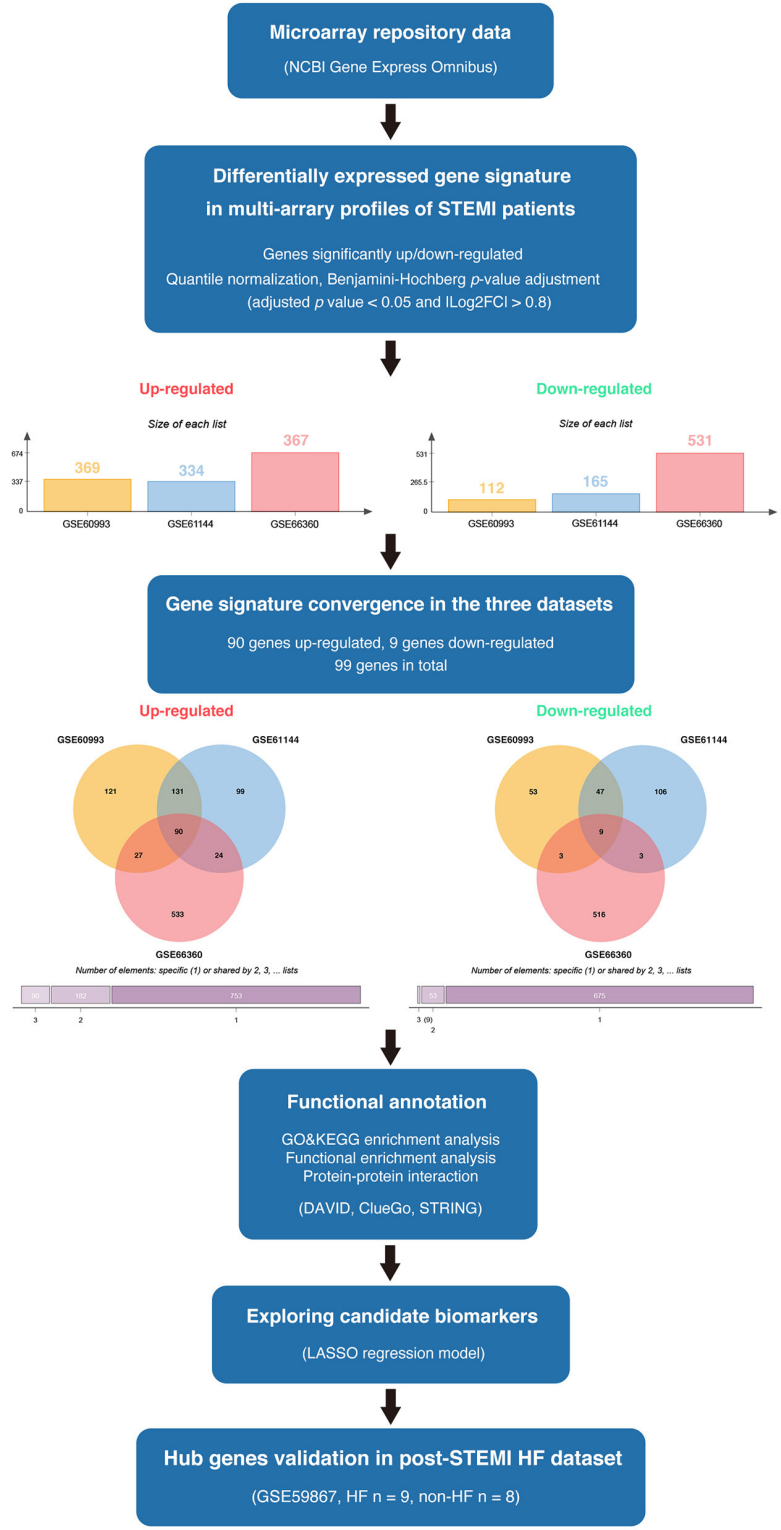
The overview of the analysis procedure. We downloaded GSE60993, GSE61144, and GSE66360 from the NCBI-GEO database and identified 90 upregulated DEGs and nine downregulated DEGs convergence in the three datasets (|log_2_FC| ≥ 0.8 and adjusted *p* < 0.05). Gene ontology and pathway enrichment were performed *via* ClueGO (version 2.5.7), CluePedia (version 1.5.7), and the DAVID database. A protein interaction network was constructed *via* STRING. Enriched hub genes were analyzed by Cytoscape software. The logistic LASSO regression was performed to build a machine learning model. GSE59867 dataset was utilized to validate the hub genes in patients with post-STEMI HF.

The available numerical expression values of patients with STEMI and healthy controls from GSE60993, GSE61144, and GSE66360 were used to identify DEGs. As shown in Figure 1, compared with the control samples, there were 369, 334, 367 upregulated and 112, 165, 531 downregulated DEGs in the patients with STEMI of GSE60993, GSE61144, and GSE66360, respectively (|log_2_FC| ≥ 0.8, adjusted *p* < 0.05, and detailed expression data with gene symbols are listed in Supplementary Tables S1–S3). Owing to the different sources of the samples, we collected the DEGs signature convergence in the three datasets to obtain the common genomic variances, which could be effective in avoiding bias and selecting solid results. Ninety-nine common DEGs, including 90 upregulated and nine downregulated, in the three datasets are summarized in Table 1, and the number of elements shared by each dataset is shown in Figure 1.

**Table 1:** The DEGs convergence in GSE60093, GSE61144, and GSE66360 in the comparison of STEMI patients with healthy controls.

	**DEGs**
Up-regulated DEGs (*n* = 90)	*TMCC3, SLC22A4, AQP9, CSF3R, DIRC2, ANXA3, CD55, DYSF, CLEC4E, SLC2A3, NCF2, SIPA1L2, PILRA, TM6SF1, NFIL3, GCA, CYP4F3, MCEMP1, BST1, CEBPD, LILRA2, CPD, TLR8, PHC2, OSM, ARG1, DUSP1, IRAK3, SULF2, PFKFB3, DAPK2, IRS2, CSNK1D, TRIB1, RBP7, BCL6, MME, QPCT, PELI1, GAB2, SLC11A1, KIF1B, IFRD1, LILRA5, CREB5, FPR1, VNN3, ITGAM, RGS2, MGAM, FCGR2A, OSCAR, CLEC4D, ABHD5, CMTM2, GPR97, PYGL, PLXDC2, LILRB3, SPI1, PADI4, IL1R2, ACSL1, ANPEP, PANX2, TLR4, ENTPD1, EMR3, SIGLEC5, LAMP2, MMP25, LRG1, MXD1, CSF2RA, MANSC1, RNF130, BMX, S100A12, TREM1, TLR2, CEBPB, VNN2, KCNJ15, CXCL16, CDA, CRISPLD2, PLAUR, PGD, STK17B, MMP9*
Down-regulated DEGs (*n* = 9)	*SAMD3, GZMA, GZMK, SBK1, NCR3, CD2, KLRG1, PTPN4, EOMES*

*DEGs were set as |log_2_FC| ≥ 0.8 and adjusted p <0.05*.

### Functional Annotation and Enrichment of DEGs

**Figure 2:**
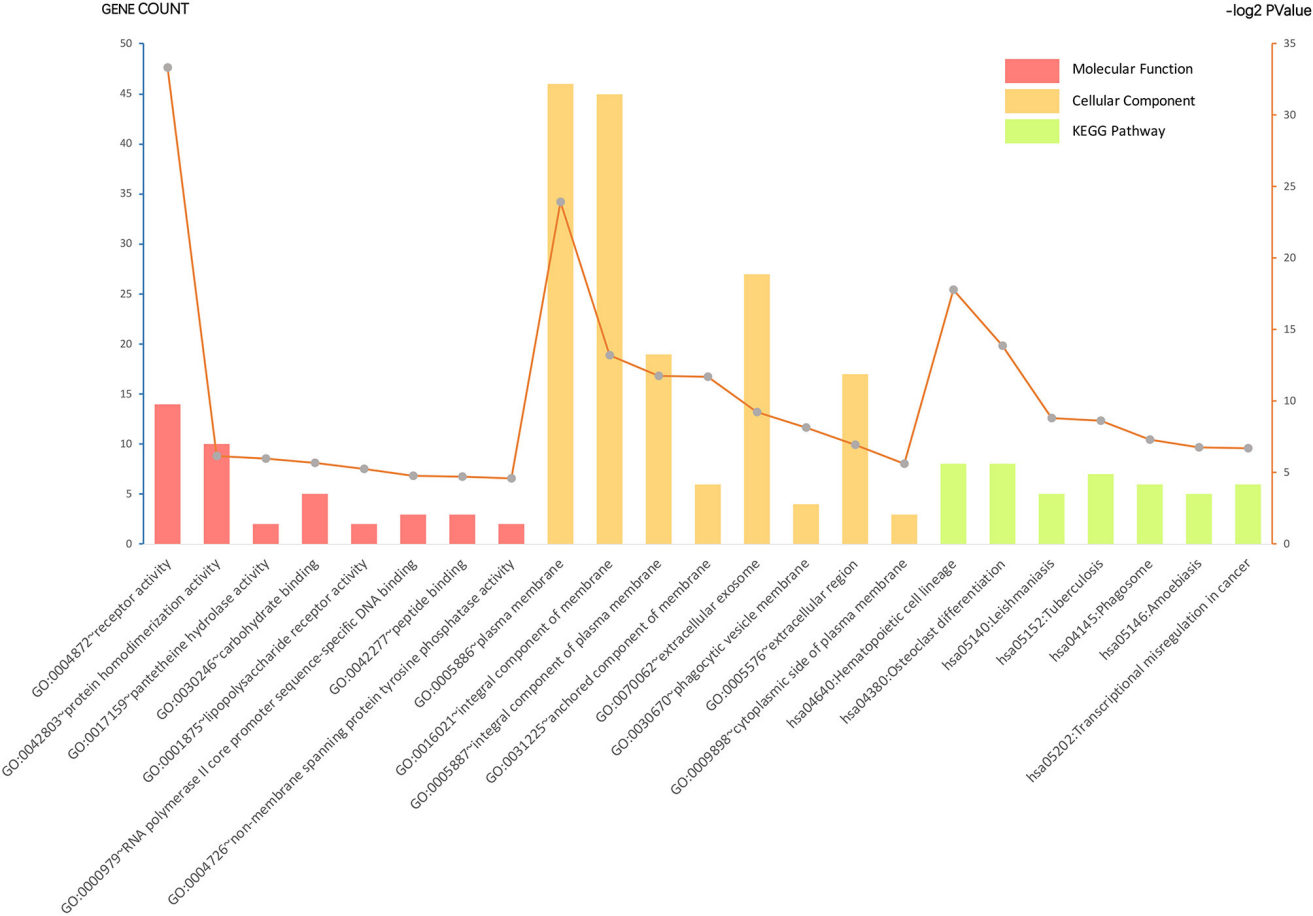
Gene ontology (GO) analysis and significant enrichment of the DEGs. GO analysis classified selected genes into the cellular component (CC), molecular function (MF), and KEGG pathway group, ranking significant enriched GO terms of the DEGs. The vertical axis on the left and the bar plot represents the gene count per term, and the vertical axis on the right and the gray dots represent log2 *p*-value (please consult Supplementary Table S4 for details). A *p* < 0.05 was considered statistically significant.

Gene ontology analysis of the common DEGs was conducted *via* the DAVID database, as well as ClueGO and CluePedia tool kits in Cytoscape. The significantly enriched molecular function, cellular component, and KEGG pathway items were selected and are shown in Figure 2 with the *p*-value. The five most enriched molecular function annotations were: (i) “GO:0004872~receptor activity” (*p* = 2.68E−28); (ii) “GO:0042803~protein homodimerization activity” (*p* = 0.014); (iii) “GO:0017159~pantetheine hydrolase activity” (*p* = 0.016); (iv) “GO:0030246~carbohydrate binding” (*p* = 0.019); and (v) “GO:0001875~lipopolysaccharide receptor activity” (*p* = 0.026), containing 14, 10, two, five, and two DEGs from the query set, respectively. Other highly enriched BP annotations included “GO:0006954~inflammatory response,” “GO:0050776~regulation of the immune response,” and “positive regulation of signal transduction.” For the analysis of enriched KEGG pathway annotations, the five most significantly enriched pathways were: (i) “hsa04640:Hematopoietic cell lineage” (*p* = 4.44E−6); (ii) “hsa04380:Osteoclast differentiation” (*p* = 6.50E−5); (iii) “hsa05140:Leishmaniasis” (*p* = 0.002); (iv) “hsa05152:Tuberculosis” (*p* = 0.002); and (v) “hsa04145:Phagosome” (*p* = 0.006), containing eight, eight, five, seven, and six genes from the query set, respectively. The outcomes of the cellular component analysis are listed in Figure 2 with specific items and *p*-value. Please consult Supplementary Table S4 for detailed information.

**Figure 3:**
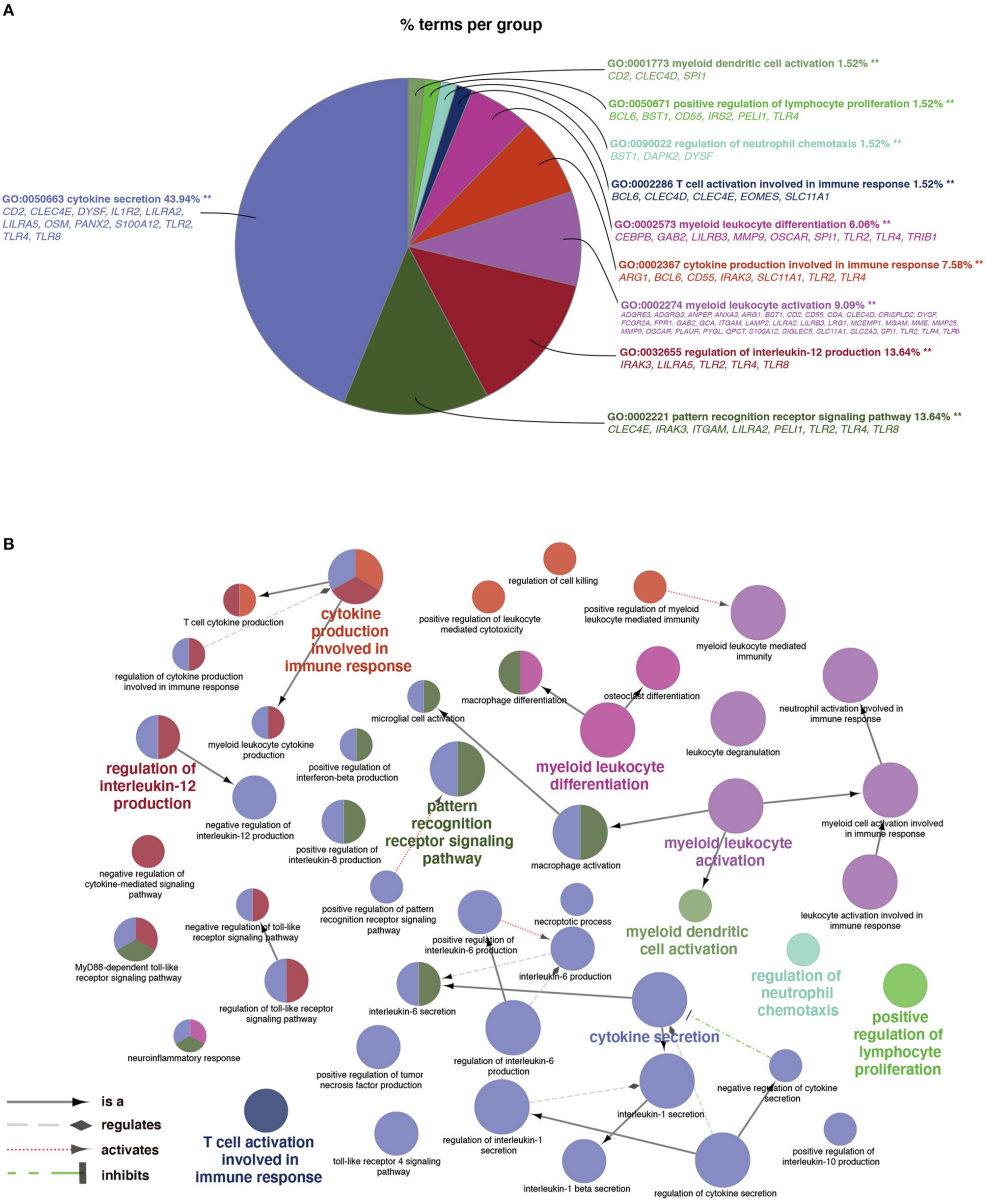
Terms of biological process (BP) by GO analysis. **(A)** Representative functional BP groups selected by the hypergeometric test and the percentage of terms per group. **(B)** The ontology relations of the annotated terms. A *p* < 0.05 was considered statistically significant.

As shown in Figure 3A, a total of 66 significant BP terms (*p* < 0.05, refer to Supplementary Table S5 for details) were classified into 10 groups according to the Cohen's kappa score based on shared genes between the terms (25). The leading group terms based on the highest significance and the percentages of terms per groups were (1) GO:0050663~cytokine secretion (*p* = 1.97E−8, 43.94%); (2) GO:0002221~pattern recognition receptor signaling pathway (*p* = 9.33E−6, 13.64%); (3) GO:0032655~regulation of interleukin-12 production (*p* = 1.80E−6, 13.64%); (4) GO:0002274~ myeloid leukocyte activation (*p* = 9.82E−29, 9.09%); (5) GO:0002367~cytokine production involved in immune response (*p* = 3.32E−6, 7.58%); (6) GO:0002573~myeloid leukocyte differentiation (*p* = 2.44E−6, 6.06%); (7) GO:0002286~T cell activation involved in immune response (*p* = 2.68E−4, 1.52%); (8) GO:0090022~regulation of neutrophil chemotaxis (*p* = 9.46E−4, 1.52%); (9) GO:0050671~positive regulation of lymphocyte proliferation (*p* = 0.0001, 1.52%); and (10) GO:0001773~myeloid dendritic cell activation (*p* = 0.0005, 1.52%). Additionally, interleukins including IL-1, IL-6, IL-8, and IL-10 were also annotated with significance. The ontology relations between different GO terms are shown in Figure 3B.

### Protein Interaction and Module Analysis

**Figure 4:**
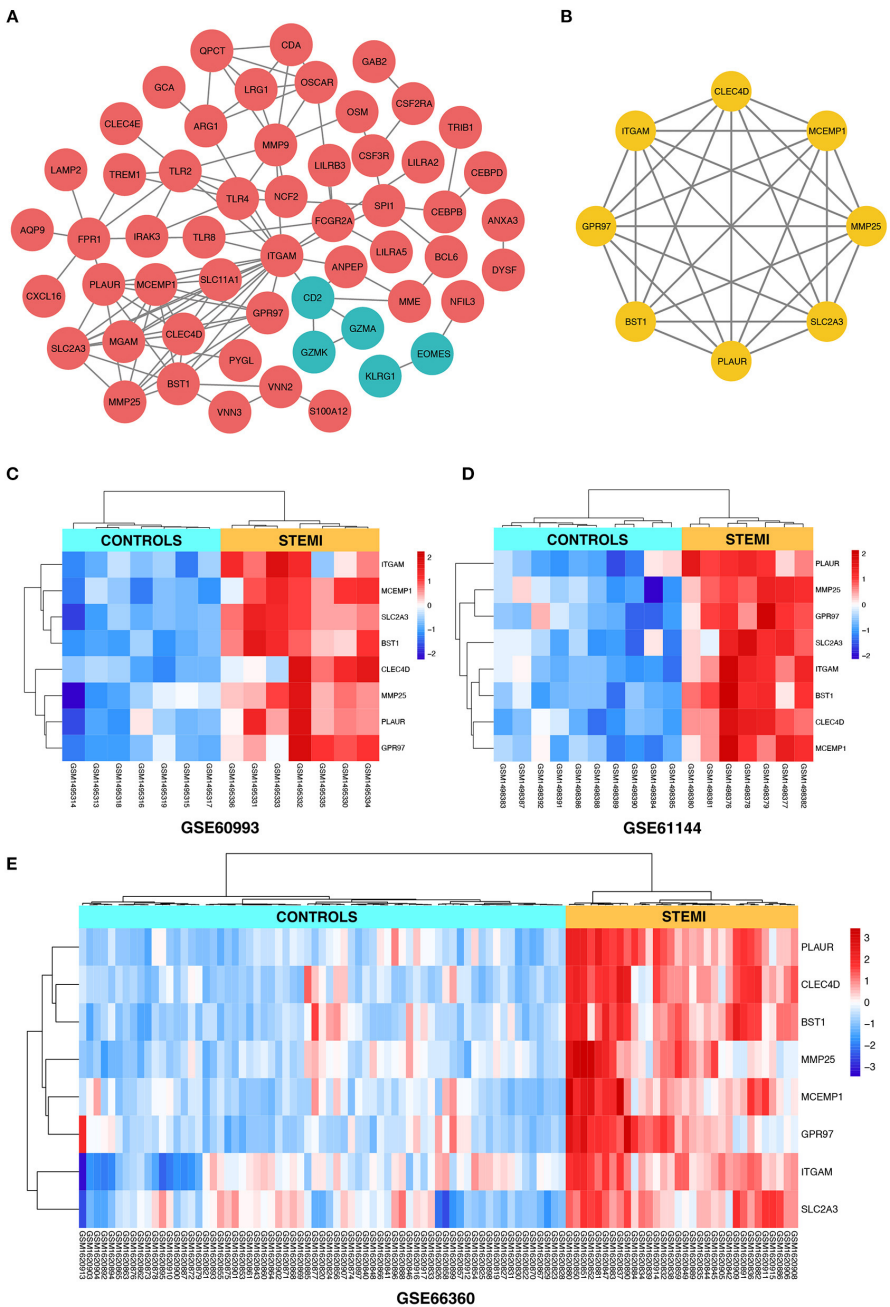
**(A)** The construction of the PPI network based on the DEGs. The red ellipse represents upregulated DEGs, the green ellipse represents downregulated DEGs. **(B)** The hub gene cluster with the highest scores in the PPI network is displayed by the yellow ellipse. **(C–E)** The heatmaps with clustering analysis showed the normalized expression values of hub genes in GSE60993, GSE61144, and GSE66360 datasets, respectively.

Search Tool for the Retrieval of Interacting Genes online database was used to reassess and predict the PPI network of DEGs, and Cytoscape software was used for the graphical representation of the network of inferred, weighted protein interactions, which provides an automated view of functional linkage, facilitating the analysis of modularity in BP es (30). Based on the high confidence level of 0.70, a total of 99 DEGs were filtered into the PPI network, and 55 nodes with 107 edges were identified, including 50 upregulated and five down-regulated DEGs (Figure 4A). The plug-in kit MCODE was conducted to analyze the significant module, and an eight-node module with 28 edges was selected from the PPI network (Figure 4B), the eight-hub genes were *ITGAM* (degree = 18), *CLEC4D* (degree = 9), *SLC2A3* (degree = 9), *BST1* (degree = 9), *MCEMP1* (degree = 9), *PLAUR* (degree = 8), *GPR97* (degree = 7), and *MMP25* (degree = 7). The detailed information and expression changes of the hub gene in each dataset are shown in Table 2. Functional annotation revealed that the eight-hub genes were all associated with the components of the plasma membrane, especially granule membrane proteins (GO:0042581 and GO:0035579, both *p* < 0.001). The normalized expression levels of the eight-hub genes in the samples from different datasets were displayed *via* the “heatmaps” package in the R platform. Parallel heatmaps with carefully designed annotation graphics are powerful for the efficient visualization of patterns and relationships among high-dimensional genomic data (35). As shown in Figure 4C (GSE60993), Figure 4D (GSE61144), and Figure 4E (GSE66360), it could be identified that the eight-hub genes were significantly upregulated in STEMI samples compared with controls, and the clustering results displayed in the heatmaps also exerted decent performance in distinguishing the samples from different conditions.

**Table 2:** Detailed information of the hub genes.

**Gene name**	**Description**	**GSE60993**		**GSE61144**		**GSE66360**	
		**log_**2**_FC**	**Adjusted *p* value**	**log_**2**_FC**	**Adjusted *p* value**	**log_**2**_FC**	**Adjusted *p* value**
*ITGAM*	Integrin subunit alpha M	0.8986	1.09E−2	0.8506	1.55E−5	0.9132	3.46E−2
*CLEC4D*	C-type lectin domain family 4 member D	1.5638	1.62E−2	1.7151	3.10E−6	2.6189	2.52E−9
*SLC2A3*	Solute carrier family 2 member 3	0.9867	4.68E−4	1.0359	3.02E−4	1.1033	3.70E−6
*BST1*	Bone marrow stromal cell antigen 1	1.1451	4.12E−4	0.9381	4.77E−6	2.0780	2.23E−8
*MCEMP1*	Mast cell expressed membrane protein 1	1.7537	9.86E−4	1.8760	2.06E−5	1.3833	4.15E−7
*PLAUR*	Plasminogen activator, urokinase receptor	1.0007	6.35E−3	0.8102	4.71E−5	2.3098	1.05E−9
*GPR97*	Adhesion G protein-coupled receptor G3	1.4664	3.55E−3	1.2396	9.35E−5	1.3983	8.66E−7
*MMP25*	Matrix metallopeptidase 25	1.1087	1.72E−2	1.5248	8.97E−5	1.2299	6.56E−6

*Description of the gene was obtained via Human Genome Resources at NCBI. The log_2_FC and adjusted p value were calculated in comparison of STEMI patients with healthy controls in the three datasets*.

### Exploring Candidate STEMI-Related DEGs by LASSO Regression and ROC Curves

**Figure 5:**
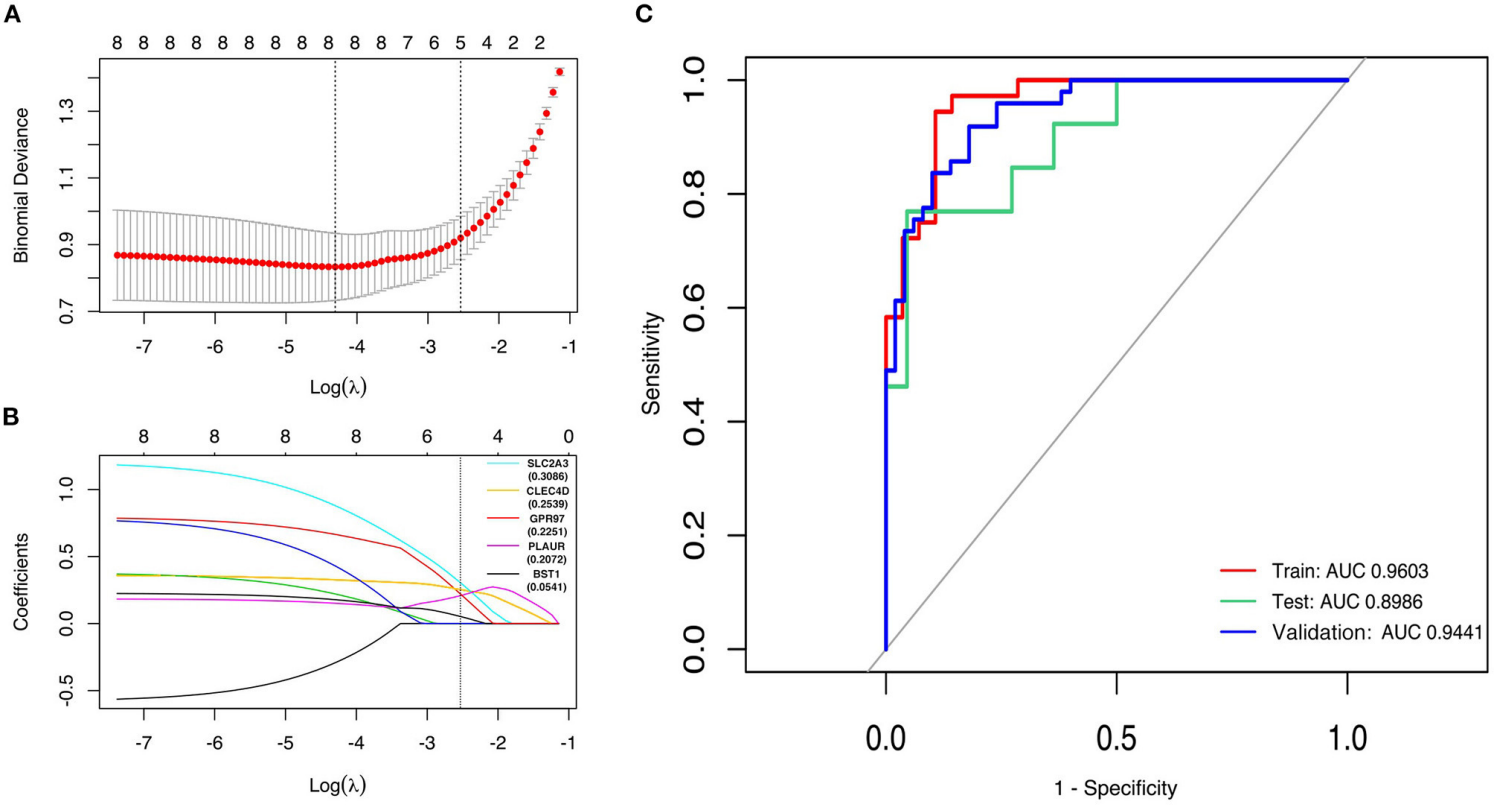
Construction of LASSO regression model and ROC curves of hub genes. **(A)** The plot indicates binomial deviance of different numbers of variables revealed by the LASSO regression model for GSE66360. The red dots represent the value of binomial deviance; the gray lines represent the standard error (SE); the vertical dotted lines represent optimal values by the minimum criteria and 1-SE criteria. “Lambda” is the tuning parameter. **(B)** The plot determines the coefficient by 1-SE criteria of LASSO regression model 0.3086, 0.2593, 0.2251, 0.2072, and 0.0541 for *SLC2A3, CLEC4D, GPR97, PLAUR*, and *BST1*, respectively. **(C)** The ROC curves of the LASSO regression model of training, testing, and 5-fold cross-validation in GSE66360.

First, the logistic LASSO regression model for the eight-hub DEGs in the comparison of STEMI with control samples from GSE60993, GSE61144, and GSE66360 was conducted to investigate an optimum linear combination in predicting STEMI (Figures 5A,B), with coefficients 0.3086, 0.2593, 0.2251, 0.2072, and 0.0541 for *SLC2A3, CLEC4D, GPR97, PLAUR*, and *BST1*, respectively. Then, the ROC curve analysis of the LASSO regression model was conducted to predict patients with STEMI in GSE66360 in the training set, testing set, and 5-fold cross-validation, with the AUC values being 0.9603, 0.8986, and 0.9441, respectively (Figure 5C), which suggested that it might have the outstanding potentiality for distinguishing the patients with STEMI from healthy controls.

### Hub Genes Validation Targeting Potential Prognostic DEGs for Post-STEMI HF

**Figure 6:**
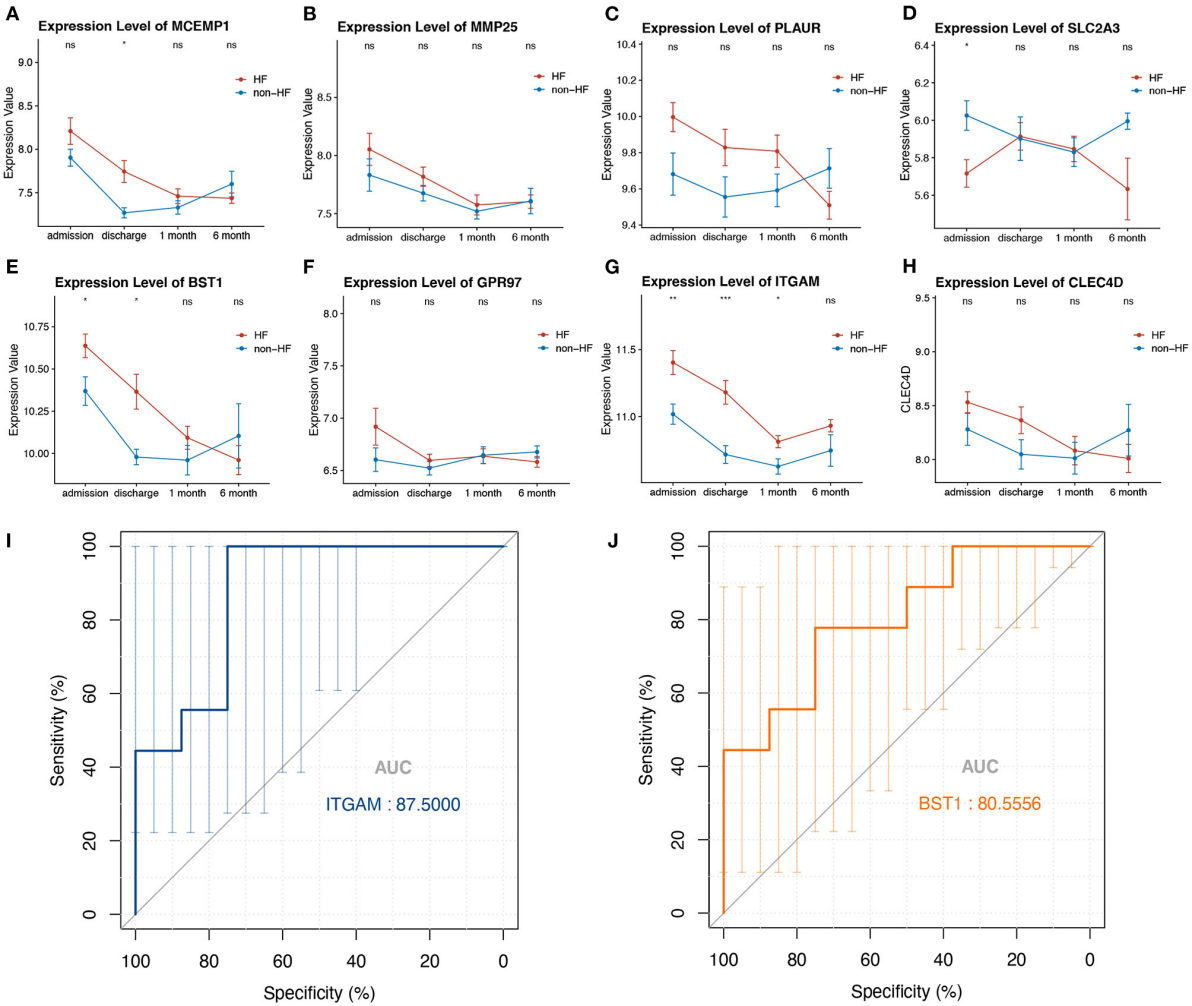
**(A–H)** Gene variations over time for investigated hub genes in patients with post-STEMI HF and patients with non-HF at different time points after STEMI (on admission, discharge, 1 month, and 6 months). Red line: HF patients, black line: patients with non-HF. Bars in high and low represent the maximum and minimum gene expression values, respectively. Statistical significance: *p < 0.05; **p < 0.01; ***p < 0.001, Wilcoxon rank-sum test. **(I,J)** ROC curves for *ITGAM*, and *BST1*, respectively. AUC, area under the curve; ROC, receiver operating characteristic. The bars represented the area of 95% CI.

To evaluate whether the eight-hub genes are differentially expressed in patients with post-STEMI HF compared with patients with non-HF or not, we investigated the expression levels of these hub genes in an external cohort from GSE59867 [patients with post-STEMI HF (*n* = 9), and patients with non-HF (*n* = 8)] with no significant gender differences (22). Interestingly, except for *SLC2A3*, seven (*n* = 7) of the hub genes in patients with post-STEMI HF were observed upregulated on the first day of STEMI (admission), especially *BST1* and *ITGAM* with significance, as compared with those who also presented with STEMI on admission but did not progress to HF during the 6 months of follow-up (Figures 6A–H). We further conducted ROC analysis to investigate the value of *BST1* and *ITGAM* as prognostic DEGs of post-STEMI HF, and the analysis showed a good predictive accuracy (Figures 6I,J). For *BST1*, the sensitivity was 77.7778% and specificity was 75% at the best cut-off value of 10.54105, with the AUC being 80.5556%; for *ITGAM*, at the best cut-off value of 11.05525, the sensitivity and specificity were 100% and 75%, respectively, and the AUC was 87.5%. Based on these results, the expression levels of *BST1* and *ITGAM* could not only differentiate patients with STEMI from healthy controls but also highly specific and sensitive DEGs for predicting post-STEMI HF.

## Discussion

As a disease that is predominantly focused in developed countries, AMI is now becoming growingly common in developing countries and encompasses a broad and heterogeneous population. More than 3 million people each year are estimated to have an acute STEMI globally (36). Diagnosis of STEMI hinges on physical and laboratory examinations, and ECG, which may be challenging, and the misinterpretation of ECG results in lower-quality care in the emergency department, yet several patients with chest pain but who do not manifest the diagnostic signs and are discharged can ultimately have dire consequences (37, 38). Meanwhile, important breakthroughs in the development of drugs and treatments have led to improved outcomes. Therefore, emerging efforts have been focused on discovering sensitive and specific DEGs to facilitate the early installment of appropriate risk stratification of patients with STEMI (39). Unfortunately, recent studies have consistently shown that inflammation-related markers of plaque instability, including myeloperoxidase and C-reactive protein, exert very low diagnostic accuracy when measured with currently available assays and therefore are not helpful in the early diagnosis of AMI (40, 41).

Transcriptome analysis enables a deep understanding of the complicated physiological processes, which has been a promising tool and applied successfully to numerous diseases including cardiovascular disorders (42, 43). From the onset of STEMI, molecular stress responses take place and are reflected by changes in gene expression, which enables peripheral blood transcriptome to be a potent predictor to discriminate the acute phase of STEMI (20). The utilization of blood as a surrogate tissue that can be collected with a minimally invasive technique is an enticing alternative to cardiac biopsy. As the requirement for specialized cell sorting samples is a barrier for diagnostic settings, we attempted to move beyond and investigate gene expression profiles that could be detected from whole blood which might facilitate the clinical feasibility *via* the real-time reverse transcription-polymerase chain reaction (RT-qPCR). Moreover, the gene signatures reported in previous studies were not robust enough, which may arise from the relatively small sample size. Accordingly, we thought it appropriate to investigate more reliable and reproducible hub genes by merging the genomic datasets from similar studies. In this study, we merged gene expression profiles in blood samples of patients with STEMI from different public datasets to investigate biologically relevant transcriptional signatures as underlying biomarkers and applied machine learning to evaluate the predictive potentiality of the selected features statistically. We further validated the expression level of hub genes in the patients who developed HF after STEMI and identified prognostic biomarkers correlated with STEMI and post-STEMI HF.

Initially, we identified 90 upregulated DEGs and nine downregulated DEGs convergence in the three selected datasets by the criteria of |log_2_FC| ≥ 0.8 and adjusted *p* < 0.05. We deliberately relaxed the restrictions to include more variant transcripts for further analysis. The GO analysis revealed the biological alterations in the pathogenesis of patients with STEMI. Noteworthy, cytokine secretion, pattern recognition receptors (PRRs) signaling pathways, and immune cells activation were the most prominent terms in the BP annotation of our study. Various cytokines have been documented to exert vital regulatory functions under the physiological and pathophysiological process of cardiac dysfunction, namely ischemic heart disease, myocardial infarction, HF, and cardiomyopathies (44–46). It is pointed out that interleukins such as IL-1, IL-4, IL-6, and IL-8 are involved in the development of myocardial infarction, most of which are released into the circulation and serve as inflammatory biomarkers (44), and these effects were also reflected in our results. On the other hand, certain cytokines such as IL-4, IL-6, IL-8, and IL-10 are also considered to exert beneficial effects from postischemic tissue repair (44). Pleiotropism of cytokine function seems to be due to the duration of the disease as well as the concentrations in the blood. It is indeed a great challenge to translate promising animal experimental data into clinical practice, and anticytokine therapies continue to require further evaluation in humans. Interestingly, regulation of IL-12 production was one of the predominant GO terms in our study. IL-12 is a proinflammatory cytokine produced by dendritic cells, macrophages, and B cells in response to microbial pathogens (47). Evidence from the literature keeps highlighting the impact of gut bacterial communities on coronary artery disease (48), and of note that bacterial DNA was detected in the human atherosclerotic plaques (49). Hence, we hypothesized that from the perspective of gut microbiota, host PRRs could modulate microbial recognition to adjust the structure and function of the mutualistic microbes, which in turn induces cytokine secretion and the downstream innate immune responses to contribute to the progression of coronary artery disease and the occurrence of STEMI (48).

We constructed the PPI network with the common DEGs shared by the three datasets, which listed the eight upregulated hub genes including *ITGAM, CLEC4D, SLC2A3, BST1, MCEMP1, PLAUR, GPR97*, and *MMP25*. Surprisingly, all these eight genes encode cellular membrane proteins which are instrumental to the delivery of synergistic outside-in signals, leading to optimal cell adhesion and migration, and inducing inflammation through damage-associated molecular patterns and pathogen-associated molecular patterns (50–52). Additionally, in concert with prior studies, some of these hub genes are reported to be associated with the pathogenesis of cardiovascular diseases. Integrin subunit alpha M (*ITGAM*) gene encodes Integrin αM (CD11b), which might promote the development and progression of abdominal aortic aneurysm *via* mediating the adhesion of endothelial cells and the transendothelial migration of circulating monocytes/macrophages (53). C-type lectin domain family 4 member D (*CLEC4D*) and mast cell expressed membrane protein 1 (*MCEMP1*) are identified to be potential prognostic and diagnostic biomarkers for ischemic stroke (54, 55). Solute carrier family 2 member 3 (*SLC2A3*) is reported to correlate with platelet aggregation (56), syndromic congenital heart disease (57), and chronic thromboembolic pulmonary hypertension (58). Although it is hard to fully elucidate the exact function of the upregulated multimolecular complex both in physiological and pathological situations, our study provided an overview of their expression and role in STEMI, which indicated their distinct properties in the disease progression. We then attempted to evaluate the predictive power of candidate genes *via* a machine learning algorithm. LASSO regression analysis was performed due to its ability to shrink coefficients of hub genes that do not contribute to the model to zero (33), and only *SLC2A3, CLEC4D, GPR97, PLAUR*, and *BST* were determined to be significantly predictive for STEMI. As demonstrated by the ROC curves, the AUC values were decent suggesting it might have the outstanding potentiality for early diagnosis of STEMI from the blood samples of patients.

Acute myocardial infarction is the underlying cause of left ventricle (LV) systolic dysfunction and HF (59). The prognosis of patients after AMI primarily depends on the degree of myocardial damage during the acute phase. Consequently, we also attempted to identify the hub genes simultaneously related to HF development in patients post-STEMI, which may be helpful in identifying DEGs of individuals at high risk for the development of HF. In the validation cohorts composed of HF and patients with non-HF post-STEMI, we observed that the long-term LV dysfunction had a similar biosignature in blood transcriptome already in the acute phase of STEMI, especially the significantly upregulated expression of *BST1* and *ITGAM* simultaneously appeared on the first day of STEMI. Additionally, the ROC analysis has indicated that these two transcripts are likely to be novel biomarkers with high sensitivity and specificity for the early prognosis of patients with post-STEMI HF. We postulate that the significant upregulation of these genes is associated with the more severe initial damage to the cardiomyocytes, culminating later in HF. To be more important, the results may also provide useful insights to look for genetic predisposition to the progression of LV remodeling and HF after STEMI.

There remain several limitations in this study. First, no information on gender was included in the datasets, and the post-STEMI HF cohort is relatively small. Additionally, the databases of GO will be revised such that our analysis may have to be repeated as the database becomes more detailed. Moreover, the results from the gene array and bioinformatic analysis require further biological proof-of-concept studies to verify. Under these conditions, large-scale and prospective investigations with strict follow-up protocols are required in the future to confirm the clinical feasibility of the proposed biomarkers detectable in whole blood for earlier identification of STEMI and prognosis of HF development.

## Conclusions

In summary, our study has the merit of assessing the integrated transcriptomic signature of patients with STEMI from several independent cohorts through reanalyzing three publicly available microarray gene expression profiling. The results reveal that STEMI is endowed with characteristic gene expression changes harboring predictive potentiality *via* machine learning, and further validation in patients with post-STEMI HF discovers prospective features for HF development. Our study offers new insights and specific biomarkers to be further explored, which might allow the early risk stratification of STEMI by simple RT-qPCR at the emergency department.

## Data Availability Statement

The datasets presented in this study can be found in online repositories. The names of the repository/repositories and accession number(s) can be found in the article/Supplementary Material.

## Author Contributions

JX and YY were involved in the conception of this study and checked the manuscript. JX analyzed the dataset and prepared the figures and tables. Both authors have read and agreed to the published version of the manuscript.

## Funding

This study was supported by the Chinese Academy of Medical Science (CAMS) Innovation Fund for Medical Sciences (CIFMS, 2016-I2M-1-009).

## Conflict of Interest

The authors declare that the research was conducted in the absence of any commercial or financial relationships that could be construed as a potential conflict of interest.

## Publisher's Note

All claims expressed in this article are solely those of the authors and do not necessarily represent those of their affiliated organizations, or those of the publisher, the editors and the reviewers. Any product that may be evaluated in this article, or claim that may be made by its manufacturer, is not guaranteed or endorsed by the publisher.
